# The Association Between Pain Relief Using Video Games and an Increase in Vagal Tone in Children With Cancer: Analytic Observational Study With a Quasi-Experimental Pre/Posttest Methodology

**DOI:** 10.2196/16013

**Published:** 2020-03-30

**Authors:** Mario Alonso Puig, Mercedes Alonso-Prieto, Jordi Miró, Raquel Torres-Luna, Diego Plaza López de Sabando, Francisco Reinoso-Barbero

**Affiliations:** 1 Juegaterapia Foundation Madrid Spain; 2 Pediatric Pain Unit Anesthesiology-Critical Care Service University La Paz Hospital Madrid Spain; 3 Department of Psychology Unit for the Study and Treatment of Pain-ALGOS Rovira i Virgili University Tarragona Spain; 4 Pediatric Hemato-oncology Service University La Paz Hospital Madrid Spain; 5 Department of Anatomy-Histology and Neuroscience School of Medicine Universidad Autónoma de Madrid Madrid Spain

**Keywords:** hematology oncology, pediatric patient, acute pain, patient-controlled analgesia, video pupilometer, analgesia nociception index

## Abstract

**Background:**

Patients with secondary pain due to mucositis after chemotherapy require treatment with morphine. Use of electronic video games (EVGs) has been shown to be an effective method of analgesia in other clinical settings.

**Objective:**

The main objective of this study was to assess the association between the use of EVGs and the intensity of pain caused by chemotherapy-induced mucositis in pediatric patients with cancer. The secondary objective was to assess the association between changes in pain intensity and sympathetic-parasympathetic balance in this sample of pediatric patients.

**Methods:**

Clinical records were compared between the day prior to the use of EVGs and the day after the use of EVGs. The variables were variations in pupil size measured using the AlgiScan video pupilometer (IDMed, Marseille, France), heart rate variability measured using the Analgesia Nociception Index (ANI) monitor (Mdoloris Medical Systems, Loos, France), intensity of pain measured using the Numerical Rating Scale (score 0-10), and self-administered morphine pump parameters.

**Results:**

Twenty patients (11 girls and nine boys; mean age 11.5 years, SD 4.5 years; mean weight 41.5 kg, SD 20.7 kg) who met all the inclusion criteria were recruited. EVGs were played for a mean of 2.3 (SD 1.3) hours per day, resulting in statistically significant changes. After playing EVGs, there was significantly lower daily morphine use (before vs after playing EVGs: 35.9 vs 28.6 µg/kg/day, *P*=.003), lower demand for additional pain relief medication (17 vs 9.6 boluses in 24 hours, *P*=.001), lower scores of incidental pain intensity (7.7 vs 5.4, *P*=.001), lower scores of resting pain (4.8 vs 3.2, *P*=.01), and higher basal parasympathetic tone as measured using the ANI monitor (61.8 vs 71.9, *P*=.009). No variation in pupil size was observed with the use of EVGs.

**Conclusions:**

The use of EVGs in pediatric patients with chemotherapy-induced mucositis has a considerable analgesic effect, which is associated physiologically with an increase in parasympathetic vagal tone despite lower consumption of morphine.

## Introduction

Pediatric patients with cancer may experience chronic pain associated with the development of the illness itself, which is caused by compression or infiltration of nearby organs or nerves or by pathologic bone fractures. However, in this population, it is very common to observe acute pain specifically associated with the diagnostic or therapeutic procedures necessary for treatment [1]. Chemotherapy-induced mucositis (International Classification of Diseases 10th Revision: K12.30; International Classification of Diseases 11th Revision: DA01.11) is a relatively common complication associated with the use of chemotherapy in the treatment of young patients with cancers, such as leukemia [2]. It is associated with intense pain, and it has a considerable impact on patient quality of life, patient mood, and the current and future evolution of the illness [3].

Acute nociception has been found to be associated with changes in the sympathetic-parasympathetic balance, including the response of pupil dilation and respiratory variations involving heart rate. A positive association has been proven between the magnitude of these vegetative changes and pain intensity [4].

Given the impact of acute pain associated with chemotherapy-induced mucositis, it is very important to administer adequate analgesia. Some of the recommended measures include lidocaine mouthwash and regulated use of analgesics, particularly strong ones such as morphine, with a patient-controlled analgesia (PCA) pump, which allows the patient to administer additional doses of morphine during peaks of intense pain [5]. However, the use of this potent opioid analgesia is not free of likely adverse effects, such as nausea or vomiting, drowsiness, itching, and urinary retention, and very serious or fatal complications, such as respiratory depression [6].

Distraction has been widely used to reduce the intensity of pain and improve function in individuals with pain, especially young individuals [7]. So-called new technologies are increasingly being used to facilitate distraction and increase effectiveness in different groups of patients with pain [8]. Electronic video games (EVGs) are of particular interest in children and adolescents owing to their proven record of attracting the attention of these individuals [9]. Although studies have shown strong positive associations between the use of EVGs and the reduction of pain intensity [10], as well as the functional adaptation of patients while undergoing different potentially painful medical interventions [7], no studies to date have proven the effectiveness of this approach for this type of pain associated with cancer-related mucositis.

Given these considerations, the main objective of this study was to assess the association between the use of EVGs and the intensity of pain (measured using the Numerical Rating Scale [NRS]), which was caused by chemotherapy-induced mucositis, in a sample of pediatric patients with cancer. As a secondary objective, the study assessed the associations of changes in pain intensity with vegetative changes in the sympathetic-parasympathetic balance assessed using a Analgesia Nociception Index (ANI) monitor (Mdoloris Medical Systems, Loos, France) and variations in pupil size assessed using AlgiScan (IDMed, Marseille, France) in this sample of pediatric patients. We hypothesized that EVG use is significantly and negatively associated with pain intensity ratings and morphine consumption. We also hypothesized that EVG use is positively associated with vagal tone.

## Methods

### Study Design

We designed an analytic observational study with a quasi-experimental pre/posttest methodology.

### Participants

Participants in this study were children and adolescents with pain secondary to mucositis, who were receiving treatment in our tertiary hospital in relation to cancer [4]. In order to take part in this study, potential participants had to provide approval. In addition, written informed consent was requested from their parents. We excluded children complaining of pain prior to mucositis. Finally, potential participants could not have any intellectual disability that might interfere with the correct use of video games.

The study included all patients aged between 4 and 17 years, who were hospitalized at the children’s oncology ward of the hospital and who received treatment between January 2016 and December 2017 for acute pain caused by grade 3 or 4 mucositis in the context of treatment for cancer [2]. According to the World Health Organization, the grades of mucositis are as follows: grade 1, soreness with or without erythema; grade 2, erythema ulcers (patient can consume a solid diet); grade 3, ulcers and extensive erythema (patient cannot consume a solid diet); and grade 4, mucositis to the extent that alimentation is not possible. Grades 3 and 4 are considered to indicate severe mucositis. Children with an intellectual inability to understand video games and those who refused to play them were excluded from the study.

### Procedure

Potential participants were invited when a PCA pump (model Sapphire; Hospira, Illinois, USA) with morphine for pain relief was indicated. At that point, the goals of the study were explained. Patients were included in the treatment protocol for acute pain at the children’s pain service, which involved a PCA pump that administered morphine chloride (1 mg morphine/kg body weight up to a maximum of 50 mg, diluted in 100 mL of saline) with the following parameters: continuous perfusion at 0-1 mL/h and a bolus on demand of 1-2 mL every 5 minutes, up to a maximum of 10 mL in 4 hours.

If a patient was under 12 years old, one of the parents or guardians was considered responsible for signing the consent form.

Those interested in taking part were offered the possibility of playing video games on PlayStation Vita (Sony Interactive Entertainment España, Madrid, Spain) on demand, with a range of different alternatives available depending on the patient’s preference and age as follows: age-rated puzzles, sports, platforms, and strategy games.

**Figure 1:**
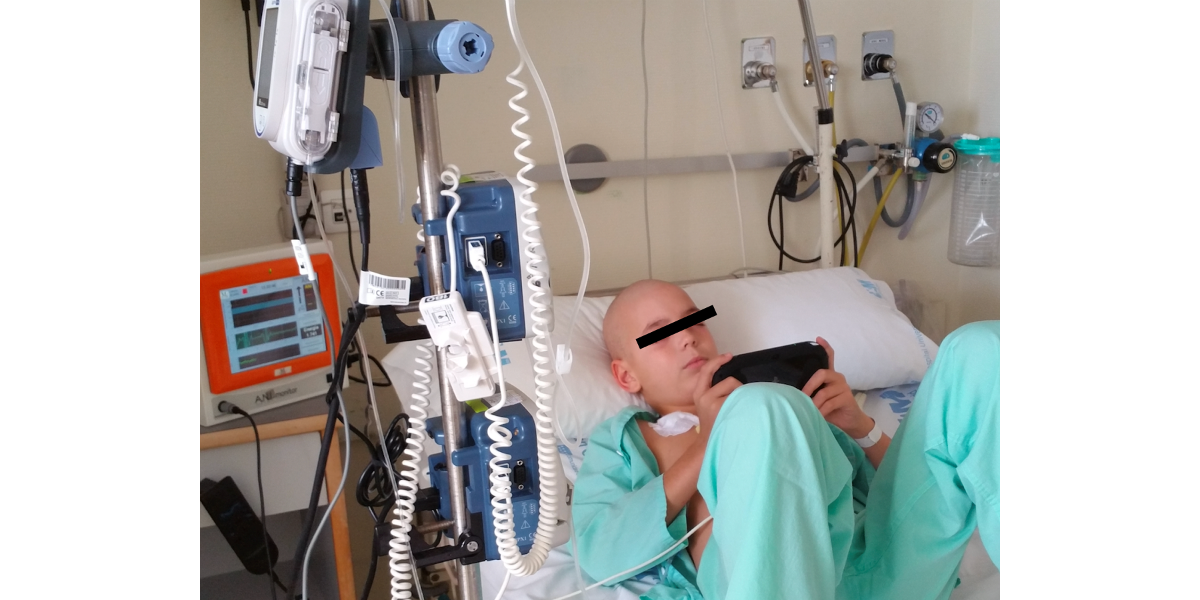
Child playing an electronic video game while being fitted with a patient-controlled analgesia pump and monitored for respiratory heart rate variability.

After 24 hours of established analgesia, patients were usually visited by members of the children’s pain service at 9 am for 30 minutes to compile information on the variables adopted (ie, pain and anxiety intensities, PCA parameters, and ANI and AlgiScan values). Figure 1 shows one of the participants with a PCA morphine pump while playing an EVG. The information on outcome variables was again collected during a visit at the same time on the following day after having played the EVG.

The study was approved by the Ethics Committee at La Paz University Hospital on May 21, 2015 (code: PI-1217).

### Measures

With regard to demographic and descriptive variables, information on age and sex was collected for descriptive purposes.

Participants were asked to report their pain intensity using the NRS-11 (scores 0-10). Participants were asked to rate their average pain intensity during the past 24 hours by choosing a single whole number between 0 (no pain) and 10 (pain as bad as could be), which best represented the pain during most of the day under rest conditions (basal or resting pain) and especially when patients tried to swallow even their own saliva (incidental pain). The NRS-11 has been found to provide valid and reliable scores when used in young people, including children as young as 6 years of age [11]. We eventually included a 4-year-old girl because she was very co-operative and understood the NRS-11 perfectly (we compared her NRS score with the score of an observational pain scale validated for her age and noted the same score).

In addition to this information, we developed a survey to collect information about the following: (1) PCA use in the previous 24 hours (information about the total morphine dose administered and the required bolus/administered bolus); (2) ANI parameters (where 100 indicates maximum parasympathetic predominance and 0 indicates maximum sympathetic predominance; maximum, minimum, and mean values during the 30-minute visit); and (3) parameters for pupil dilation (in mm) according to the pupillometry index at three time points (beginning of the visit, after 15 minutes, and end of the visit).

Finally, information was collected about the amount of time spent playing EVGs, with children’s relatives asked to report the daily total minutes played.

### Data Analysis Plan

We first calculated percentages, means, and SDs of the study variables for descriptive purposes. A Wilcoxon test was performed for categorical variables (ie, pain scores), and a Student *t* test was performed for ordinal variables (ie, dose of morphine, number of boluses required, ANI score, and pupil size).

## Results

### Sample Description

Of 25 patients invited to join the study, 20 (80%) agreed to participate in the study. Of these 20 patients, 12 had acute lymphoblastic leukemia, five had received a bone marrow transplant, and the remaining three had acute myeloid leukemia. One 6-year-old girl showed clinical signs of probable septic shock that required admission to the pediatric intensive care unit, and she was excluded from the study.

Of the remaining 19 patients, 10 were girls and nine were boys, with a mean age of 11.5 (SD 4.5; range 4-17) years. EVGs were played for a mean of 2.3 (SD 1.3) hours per day. Table 1 shows the other descriptive details and the study variables.

**Table 1:** Parameters of the patient-controlled analgesia pump, Analgesia Nociception Index monitor, and AlgiScan monitor on treatment days.

Variable	Without video games, mean (SD)	With video games, mean (SD)	Student *t* test (*t*_1,18_)	*P* value
Daily total dose of morphine (µg/kg)	35.9 (27.0)	28.6 (27.1)	3.51	.003
Number of required boluses of morphine	17 (14.9)	9.5 (9.5)	2.87	.01
ANI^a^, medial	62.1 (12.4)	71.9 (14.8)	−3.01	.009
ANI, maximum	79.6 (12.6)	86.0 (9.4)	−2.01	.06
ANI, minimum	48.2 (9.6)	55.7 (14.6)	−2.20	.045
Pupil size (mm)	4.9 (0.3)	5.3 (0.5)	−0.37	.37

^a^ANI: Analgesia Nociception Index.

### Effects of the Use of Electronic Video Games on Subjective Variables

**Figure 2:**
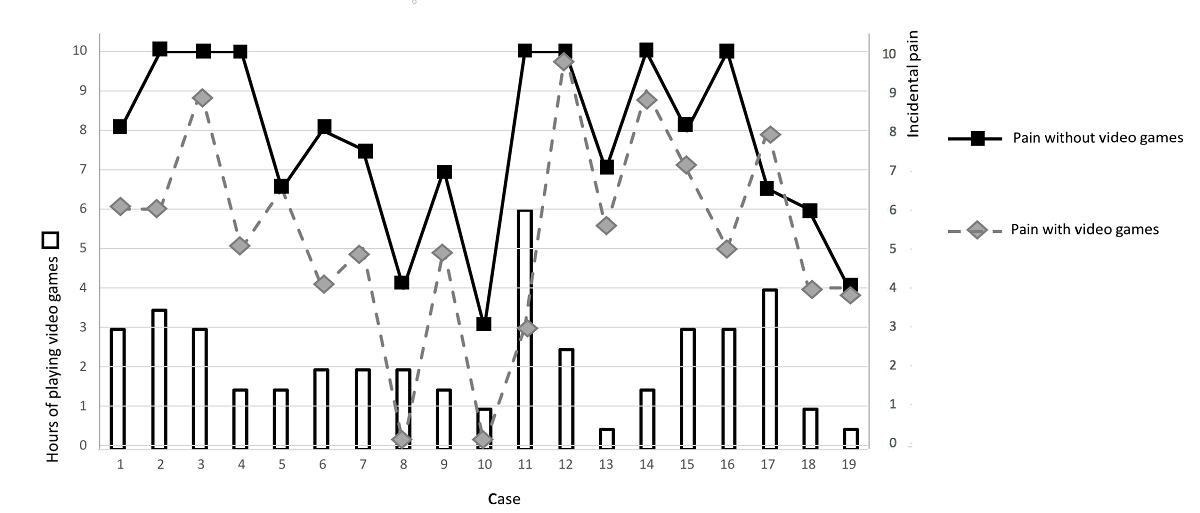
Distribution of hours of playing video games with the maximum variation in incidental pain with and without the use of video games in each patient.

**Figure 3:**
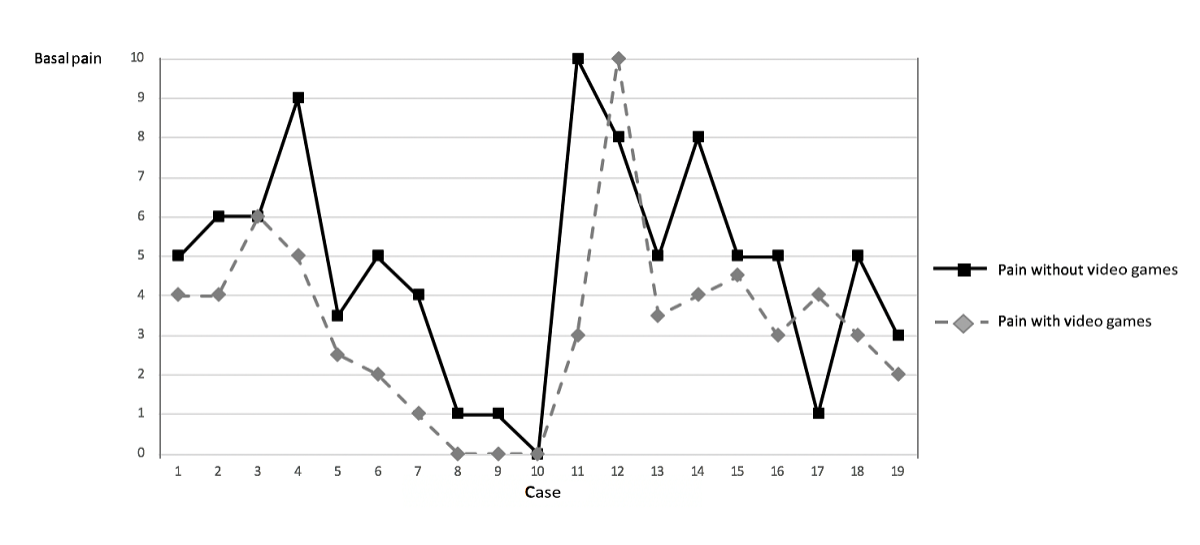
Variations in basal daily pain with and without the use of video games in each patient.

The results of the completed analyses showed that the mean incidental pain intensity reported in the previous 24 hours reduced significantly after playing EVGs (mean score with vs without EVGs: 5.4, SD 2.7 vs 7.7, SD 2.3; Z=−3.189; *P*=.001) (Figure 2). Similarly, the mean basal pain intensity reported in the previous 24 hours reduced significantly after playing EVGs (mean score with vs without EVGs: 3.2, SD 2.4 vs 4.8, SD 2.8; Z=−2.570; *P*=.01) (Figure 3).

### Effects of the Use of Electronic Video Games on Objective Variables

The parameters of the electronic PCA pump showed a 20% decrease in the daily consumption of morphine on the day the video games were played (mean value [µg/kg] with vs without EVGs: 28.6, SD 27.1 vs 35.9, SD 27.0; *P*=.003), with a 44% decrease in the number of on-demand boluses (mean number with vs without EVGs: 9.5, SD 9.5 vs 17, SD 14.9; *P*=.01).

The ANI parameters showed a statistically significant 14% increase in parasympathetic baseline tone on the day after playing the video games (mean value with vs without EVGs: 71.9, SD 14.8 vs 62.1, SD 12.4; *P*=.009) (Table 1). However, pupil size showed no changes in sympathetic tone between the two days (Table 1).

## Discussion

To the best of our knowledge, this is the first clinical trial to study the efficacy of playing video games to reduce the pain associated with chemotherapy-induced mucositis related to the treatment of young patients with cancer. The results were as expected. This is to say that as hypothesized, participants reported lower levels of pain intensity after playing video games (significant reduction by 30% regarding incidental pain and 33% regarding basal pain; Figure 3). Furthermore, the results showed that this analgesic effect was accompanied by a vegetative association involving an increase in the parasympathetic tone of the autonomic nervous system. To date, this vegetative influence had not been studied in the management of acute pain (with a duration of a few days) in oncology or other fields.

The EVGs were especially effective in reducing incidental pain that is characteristically associated with mucositis (eg, when patients are not able to even swallow saliva), and we observed a highly remarkable reduction in the peak of pain, along with a 44% decrease in the need for additional morphine boluses to control incidental pain, resulting in a 20% decrease in the daily total dose of morphine administered. This is of particular importance as a reduction in the dose of morphine would logically result in a reduction in common morphine-associated side effects, such as paralytic ileus, nausea or vomiting, and pruritus [6].

Interestingly, the effect was so intense that it influenced the sympathetic-parasympathetic balance of the patients. A sympathetic-parasympathetic imbalance is caused by the presence of a harmful stimulus that tends toward sympathetic activation, while physiological recovery is achieved by the parasympathetic system [12].

The patients included in this study had secondary pain caused by mucositis. Chemotherapy-induced mucositis causes acute pain with a high intensity regarding pain sensation [13], as can be seen from the high initial pain levels reported by our patients, despite the regulated use of intravenous analgesia. In the case of our patients’ mucositis, their pain could occasionally exacerbate, leading to very intense pain and great suffering, as well as emotional stress for their relatives [2]. This condition could also have a negative effect on our patients’ prognosis, as it could be a source of infectious complications, such as sepsis, which was experienced by one of the patients who had consented to participate but required admission to the intensive care unit.

Our results are consistent with evidence-based medicine, which has shown that EVGs are effective in pediatric patients as both psychological therapy and treatment for boosting physical function [14]. In fact, psychologically, EVGs have been shown to reduce the anxiety associated with hospitalization or cause a state similar to mental relaxation in children [15]. Furthermore, games with an interactive element have been used to encourage physical exercise and thus reduce chronic pain in children with juvenile arthritis [16].

As analgesia, video games have been proven to be useful in reducing pain among children, especially in the case of pain associated with procedures for venipuncture [7], wound cleaning and dressing changes in burn victims [10], and other painful procedures related to other chronic illnesses [17].

The mean duration of video game use in this study was close to 3 hours per day, which is the value established in several studies as the threshold after which video games start to be more harmful than beneficial to health [18]. Thus, the general recommendation to not exceed this time of 3 hours a day of playing video games in hospitalized children should be considered by both parents and responsible physicians.

Video games have already been shown to be effective in pediatric oncology patients, encouraging physical rehabilitation during the patient’s recovery phase after disease remission [19] and allowing relief from pain and anxiety associated with the placement of percutaneous central catheters [20]. As of now and according to the findings of this study, it is possible to recommend video games during the acute phase of painful oncological mucositis.

The ANI monitor measures parasympathetic activity through the analysis of respiratory sinus arrhythmia, assessing heart rate variability induced by each respiratory cycle (spontaneous or artificial). In conscious patients, the ANI indicates acute pain and stress levels. Normal values are higher than 50, and higher levels indicate higher parasympathetic-sympathetic balance. This device has been used successfully to measure the level of nociception during different painful situations, including surgery in children [21]. However, in this study, a 14% increase in the ANI was noted during the visit on the day after playing video games, suggesting that the beneficial effects of video games did not have a short duration but extended for several hours. However, no difference in pupil size was found despite the fact that pupil size directly depends on the opiate dose [22]. In this study, there was a 20% reduction in the dose of morphine, but this was not accompanied by an increase in pupil size. This finding might be associated with a high parasympathetic tone (confirmed by the ANI), which promotes pupil myosis and probably compensates the effect of a reduction in the opiate dose.

If the results of this study are confirmed in future work, the clinical implications of the findings would be great, as EVGs could be included as part of the nonpharmacological treatment plan for cancer-related mucositis in pediatric patients.

This study has some limitations that should be considered when interpreting the results. First, the sample size was small. Additional research with a larger sample size would be needed to help determine the reliability of the findings. Second, although the study design allowed for the evaluation of concurrent associations among the scores of the study variables, we were not able to test for causal associations. Thus, clinical trials with control groups are needed to evaluate the causal influence of the use of video games on outcome variables.

Despite the limitations of this study, the findings provide important additional information on the potential value of using video games to reduce pain and suffering, as well as enhance health recovery by increasing vagal tone in a very safe and efficient way. The use of video games for a mean of approximately 2 hours in children with intense mucositis relieved their pain by 30%, with a 14% increase in vagal tone, and at the same time, it reduced the daily dose of morphine by 20%.

The findings are consistent with a biopsychosocial model of pain, supporting the use of medical or physical and psychosocial interventions to prevent or manage pain in young patients [23,24]. Additional research to identify the best methods to provide this integrated help to young patients undergoing painful medical procedures is warranted.

## Abbreviations

ANI: Analgesia Nociception Index
EVG: electronic video game
NRS: Numerical Rating Scale
PCA: patient-controlled analgesia
