# Authorship Correction: The Association Between Pain Relief Using Video Games and an Increase in Vagal Tone in Children With Cancer: Analytic Observational Study With a Quasi-Experimental Pre/Posttest Methodology

**DOI:** 10.2196/19961

**Published:** 2020-07-07

**Authors:** Mario Alonso Puig, Mercedes Alonso-Prieto, Jordi Miró, Raquel Torres-Luna, Diego Plaza López de Sabando, Francisco Reinoso-Barbero

**Affiliations:** 1 Juegaterapia Foundation Madrid Spain; 2 Pediatric Pain Unit Anesthesiology-Critical Care Service University La Paz Hospital Madrid Spain; 3 Department of Psychology Unit for the Study and Treatment of Pain-ALGOS Rovira i Virgili University Tarragona Spain; 4 Pediatric Hemato-oncology Service University La Paz Hospital Madrid Spain; 5 Department of Anatomy-Histology and Neuroscience School of Medicine Universidad Autónoma de Madrid Madrid Spain

In the paper “The Association Between Pain Relief Using Video Games and an Increase in Vagal Tone in Children With Cancer: Analytic Observational Study With a Quasi-Experimental Pre/Posttest Methodology” (J Med Internet Res 2020;22(3):e16013) the authors noticed that one of the authors was not listed on the original published manuscript. The missing author was Mario Alonso Puig; they are first author on the corrected manuscript.

Mario Alonso Puig’s affiliation will be listed as “Juegaterapia Foundation, Madrid, Spain” and will appear as affiliation 1 in the corrected manuscript. Author affiliations 1-4 in the original published manuscript will be renumbered to affiliations 2-5 in the corrected manuscript.

The correction will appear in the online version of the paper on the JMIR website on July 7, 2020, together with the publication of this correction notice. Because this was made after submission to PubMed, PubMed Central, and other full-text repositories, the corrected article has also been resubmitted to those repositories.

